# Healthy bone tissue homeostasis

**DOI:** 10.1038/s12276-020-0472-3

**Published:** 2020-08-13

**Authors:** Je-Yong Choi

**Affiliations:** Department of Biochemistry and Cell Biology, School of Medicine, Bone Disease Analysis Center, Korea Mouse Phenotyping Consortium, Kyungpook National University, Daegu, 41644 Republic of Korea

Over the past 20 years, bone tissue has been studied as an endocrine organ that regulates the homeostasis of mineral ions^1^, the bone marrow niche for hematopoiesis^2^, energy metabolism^3^, and even brain function^4^. As the elderly population has grown to an unprecedented level, new interest in and consideration of healthy bone tissue homeostasis have arisen^5^. In particular, patients with osteoporosis and osteoarthritis are increasing, and many societies must seriously reevaluate their healthcare systems. In other words, people are increasingly interested in maintaining healthy bones and homeostasis along with healthy life expectancy.

In this special issue of *Experimental and Molecular Medicine*, we highlight research on how bone tissue develops during embryonic development^6^ and explore recent treatments for skeletal genetic disorders through the functional regulation of RUNX2, a key transcription factor that forms bones^7,8^. Since one of the main causes of bone tissue diseases is associated with inflammatory cytokines, also featured herein is a study of the physiological and pathological signaling pathways of these cytokines in relation to bone tissue homeostasis^9^. Finally, we highlight the causes and mechanisms of another senile joint disease, osteoarthritis, and explore the possibility of maintaining the homeostasis of the bone joint with the trace element selenium^10^. Finding ways to maintain healthy bone tissue homeostasis from various perspectives is one of the pressing challenges in medicine. We hope that the review articles presented in this special issue are informative and useful to our understanding of bone tissue homeostasis.
